# Atosiban interacts with growth hormones as adjuvants in frozen-thawed embryo transfer cycles

**DOI:** 10.3389/fendo.2024.1380778

**Published:** 2024-05-22

**Authors:** Haixiao Chen, Jiali Cai, Xiaohua Sun, Lanlan Liu, Zhenfang Liu, Peng Gao, Xiaoming Jiang, Jianzhi Ren

**Affiliations:** ^1^ Reproductive Medicine Center, Xiamen University Affiliated Chenggong Hospital, Xiamen, Fujian, China; ^2^ Medical College, Xiamen University, Xiamen, Fujian, China; ^3^ Quality Management Department, Xiamen University Affiliated Chenggong Hospital, Xiamen, Fujian, China

**Keywords:** atosiban, growth hormone, frozen-thawed embryo transfer, clinical pregnancy rate, interaction, assisted reproductive technologies

## Abstract

**Objective:**

To investigate the interaction between atosiban and growth hormone (GH) as adjuvants in frozen-thawed embryo transfer (FET) cycles

**Method:**

A total of 11627 patients who underwent FET at Xiamen University Affiliated Chenggong Hospital between January 2018 to December 2022 were retrospectively analyzed. Among them, 482 patients received atosiban and 275 patients received GH. The interactions were estimated by comparing the odds ratio (OR) for pregnancy comparing patients with or without atosiban adjuvant in cohorts stratified according to the presence of GH use in either the overall cohort or a propensity score (PS) matched cohort. An interaction term (atosiban × GH) was introduced to a multivariate model to calculate the ratio of OR (ORR) adjusted for confounders.

**Results:**

For all patients receiving atosiban administration, no obvious effect on pregnancy was observed in comparison with either matched or unmatched controls. However, when the patients were stratified according to GH administration, atosiban showed a significant association with clinical pregnancy in comparison with either matched or unmatched controls among patients with GH treatment with rate ratios (RR) of 1.32 (95%CI: 1.05,1.67) and 1.35 (95%CI: 1,1.82), respectively. On the other hand, however, the association was absent among patients without GH treatment. The adjusted ORRs in both matched and unmatched cohorts were 2.44 (95%CI: 1.07,5.84) and 1.95 (95%CI: 1.05, 3.49) respectively.

**Conclusion:**

The combination use of atosiban and GH in FET cycles is potentially beneficial to the pregnancy. However, indications for the use of atosiban and GH may need further assessment.

## Introduction

A successful assisted reproductive technology (ART) program is determined by various factors including the quality of the embryos, the receptivity of the endometrium, and the synchronized development of the embryo. Among these factors, endometrial receptivity plays a crucial role, especially when high-quality euploid embryos are transferred and fail to implant (1). Since pregnancy rates have generally plateaued following decades of improvement of ART, physicians often tend to use various add-ons or adjuvants in attempts to improve the factors relevant to implantation, even though robust evidence for these adjuvant therapies remained limited (2). Despite the criticism of unproven efficacy and additional resource consumption (3), the use of add-ons/adjuvants in ART is widespread. It is estimated that 65% of women undergoing IVF in the U.K. used one or more add-ons in 2021 (4). A survey from Australia may say the proportion of patients receiving at least 1 one-add was 82% (5). It may warrant more evidence to justify the widespread use of ART adjuvants.

While the evidence available for ART adjuvants was mainly focused on single adjuvants, patients often receive more than one adjuvant or add-ons in real-world clinical practice. A national survey showed that the median number of add-ons the patients took as part of their treatment was two (5). Sometimes, the motives for using adjuvants in ART treatments go further than technical issues (3). Driven by complex motives, such as pursuing solutions to uncertainty, combinations of adjuvants that are supposed to be helpful are used in the same cycle. However, it may further increase the uncertainty if the knowledge of the interaction between these adjuvants is lacking.

Atosiban is one of the few adjuvants that showed a trend toward increased clinical pregnancy rate in embryo transfer (ET) (2) with a supposed mechanism in antagonizing the oxytocin receptors and reducing the uterine contractions that might dislodge the transferred embryos. It also provided an option of adjuvants in patients with repeated embryo implantation failure (RIF) (6). Because the majority of RIF patients have unclear etiologies, the list of adjuvants in RIF patients is rapidly growing and a combination of adjuvants targeting different mechanisms is possibly recommended for them (6). Growth hormone (GH) provides another hopeful option for RIF that may improve endometrial growth and receptivity (7). However, previous studies suggested a potential physiological interaction between GH and oxytocin signaling (8). It might also suggest a potential interaction between GH and atosiban if they were used as adjuvants in ET cycles.

Since there is litter knowledge regarding the combination use of GH and atosiban in ART patients. The present study aims to evaluate the interaction of GH and atosiban as ET adjuvants in frozen-thawed embryo transfer cycles in a retrospective cohort.

## Materials and methods

### Study subjects

A retrospective analysis was performed on patients who underwent frozen-thawed embryo transfer (FET) cycle in the Xiamen University Affiliated Chenggong Hospital in the period from January 2018 to December 2022, with 11627 cases in total. Institutional Review Board approval for this retrospective study was obtained from the Ethics Committee of the Xiamen University affiliated Chenggong Hospital. Informed consent was not necessary, because the research was based on nonidentifiable records as approved by the Ethics Committee.

The exclusion criteria included cycles canceled for any reason (n=32), cycles with missing data concerning the cycle characteristics (n=613), and cycles with twice frozen-thawed embryos (n=50). The patients receiving other add-ons or adjuvants during the FET cycles, such as endometrial receptivity test (ERT) or gynecological immunological treatment were also excluded. Patients with luteinized unruptured follicle (LUF) syndrome during the cycles were excluded due to possible disruption of luteal phase endocrine dynamics in these patients. Finally, rare and specific cases, such as carcinoma or vaginal bleeding on the day of transfer were excluded. The inclusion/exclusion criteria were detailed in a flowchart (**Supplementary Figure S1**).

### Ovarian stimulation and laboratory protocol

All patients received an agonist or antagonist protocol with the use of FSH or HMG for ovarian stimulation as previously described (9). Oocytes were recovered 34–36 hours after administration under transvaginal ultrasound guidance and were inseminated using either conventional IVF or intracytoplasmic sperm injection(ICSI). Zygotes and embryos were cultured in traditional incubators (C200, Labotect, G¨ottingen, Germany) at 37 °C, 6%CO2, and, 5%O2 with Cook IVF media (COOK MEDICAL, Bloomington, IN) and oil overlay (Vitrolife, G¨oteborg, Sweden).

The morphological criteria of embryo scoring at the cleavage stage and blastocyst stage adhered to the Istanbul consensus (10) and the Gardner scoring criteria (11). Good quality embryos were defined as Grade 1 cleavage stage embryos according to the Istanbul consensus or blastocysts with a ≧BB Gardener score.

For all cycles, a vitrification protocol, employing 15% dimethylsulphoxide, 15% ethylene glycol, and 0.6 M sucrose as cryoprotectants, was used. Embryos obtained from the patient’s fresh egg retrieval cycle are vitrified and frozen as day 3 cleavage stage embryos or day 5 or 6 blastocysts.

### The endometrial preparation

Four major types of endometrial protocols were used for endometrial preparation, including natural cycles (NC), ovulation induction cycles (OI), hormone replacement cycles (HRT), and down-regulated hormone replacement cycles (GnRHa). The NC or OI cycle is based on vaginal ultrasound monitoring, with the day of ovulation as D0, D3 as the day of cleavage embryo thawing, and D5 as the day of blastocyst thawing and transferring; the hormone replacement cycle starts with estrogenic medication for 14–20 days from the second day of menstruation or 28 days after down-regulation of the hormone replacement cycle, and the day of addition of progesterone as D0, D4 as the day of cleavage embryo thawing, and D6 as the day of blastocyst thawing and transferring. The patients who had an unexpected inadequate follicle growth in NC protocol and chose to receive additional stimulation were assigned as “other protocol”.

### Luteal support after embryo transfer

All patients were given luteal support therapy after embryo transfer. In NC cycles, dexamethasone tablets 20 mg twice daily were given. In the OI cycle, 40 mg/day of progesterone was given intramuscularly or 90 mg/day of xylenol vaginally and 20 mg of oral dextroprogesterone tablets twice a day following ovulation. HRT or GnRHa cycles were supported with estrogen continuation and addition of intramuscular progesterone 40mg/day or vaginally administered chenodexone 90mg/day and orally administered dextroprogesterone tablets 20mg twice daily after ovulation cycle with luteal support.

Serum β-HCG test was performed at D14 after embryo transfer, if β-HCG>5 U/L, it was defined as biochemical pregnancy, and if the gestational sac was seen by ultrasound at D28, it was defined as clinical pregnancy.

### Adjuvant treatment

For patients receiving atosiban, a total dose of 37.5mg of atosiban acetate (atosiban acetate, 37.5 mg/5 ml, Nanjing Haina Pharmaceutical) was administrated as an intravenous infusion which began half an hour before embryo transfer and lasted for 3 hours. For patients receiving GH, GH (recombinant human GH for injection, 4.0 IU/1.33 mg/1.0 ml/vial, Changchun Jinsai Pharmaceutical Co., Ltd.) was administered 4 IU/day intramuscularly during the preparation of the endometrium before embryo transfer.

### Statistics

Because the supposed mechanism of atosiban is facilitating embryo implantation, we evaluated clinical pregnancy as our primary outcome of interest. The interactions were estimated by comparing the odds ratio (OR) for pregnancy comparing patients with or without atosiban adjuvant in cohorts stratified according to the presence of GH use. More formally, an interaction term (atosiban × GH) was introduced to a multivariate model to calculate the ratio of OR (ORR).

To further evaluate the potential impact of the indications associated with adjuvant usage, we also screen for the potential interactions between atosiban and a series of indications, including age, previous ET attempts, endometriosis, and endometrial thickness in the multivariate models.

To minimize the confounding, the analyses were carried out in both the overall cohort and a propensity score (PS) matched cohort. In the PS-matched cohort, patients were matched according to atosiban use, and GH treatment was the primary covariate for matching. The matching was carried out in 1:1 ratio with the allowance of discarded cases.

Other confounders and covariates used for the matching and multivariate adjustment were selected based on our experience and previous knowledge with the assistance of a direct acyclic graph (DAG). The DAG was created by dagitty software (https://dagitty.net/dags.html) and shown as a supplementary figure (**Supplementary Figure S2**). The covariates include patient characteristics (age, BMI, previous ART attempts, duration of infertility, endometriosis, PCOS, female endocrine profile, and male same parameters), ovarian stimulation (protocol and starting dosage), laboratory procedures (insemination protocols, blastocyst culture, and cryopreservation), and embryo availability (the number, stage, and quality of embryos transferred). A MatchIt package in R software was used for the PS matching (12). The cobalt package (13) was used to test the balance. Standard differences (D) were calculated to evaluate the balance of the distribution of the baseline characteristics between the groups before and after PS matching. D < 0.1 was used as the threshold to indicate a negligible difference in the mean or prevalence of a covariate (14). The balance of covariates was also examined by the distribution of propensity score (distance) between matched groups (**Supplementary Figure S3**).

Subgroup analyses for patients with a thin endometrium (endometrial thickness<8mm) and patients with previous ET failure (ET order≧3) were also carried out to evaluate the effects of adjuvants and interactions in patients with specific indications.

For descriptive analyses, continuous variables were analyzed using the Wilcoxon test, and categorical variables were analyzed using the chi-square test or Fisher’s exact test, P < 0.05 was considered to be significant. All analyses were performed by using R statistic software 4.12 (15).

## Results

In this study, 11627 FET cycles were enrolled and analyzed. There were no statistically significant differences between the groups in terms of age of infertility, years of infertility, infertility factors, endometrial preparation regimen, embryo transfer regimen, mean number of embryos transferred, and endometrial thickness. Basic characteristics before and after PS matching are shown in **Table 1**.

**Table 1:** Patient characteristics and cycle parameters in the unmatched and matched cohort.

	Unmatched				matched			
	Non-Atosiban	Atosiban	P-value	*D	Non-Atosiban	Atosiban	P-value	*D
	(N=9949)	(N=482)			(N=481)	(N=481)		
**Female age, yr**				0.1348				-0.0311
Median [Q1,Q3]	32.0 [29.0,35.0]	32.0 [30.0,35.0]	0.0149		32.0 [30.0,36.0]	32.0 [30.0,35.0]	0.596	
Mean(SD)	32.2(4.19)	32.8(4.28)			32.9(4.37)	32.8(4.28)		
**Male age, yr**				0.1428				-0.0271
Median [Q1,Q3]	33.0 [30.0,37.0]	34.0 [31.0,37.0]	0.00382		34.0 [31.0,38.0]	34.0 [31.0,37.0]	0.487	
Mean(SD)	33.8(4.73)	34.5(4.67)			34.6(4.80)	34.5(4.68)		
**Parity**				-0.0197				0
0	8432 (84.8%)	418 (86.7%)	0.266		417 (86.7%)	417 (86.7%)	>0.99	
≧1	1517 (15.2%)	64 (13.3%)			64 (13.3%)	64 (13.3%)		
**AFC**				-0.0372				0.0488
Median [Q1,Q3]	11.0 [8.00,16.0]	11.0 [7.00,15.0]	0.11		11.0 [7.00,15.0]	11.0 [7.00,15.0]	0.695	
Mean(SD)	12.0(6.18)	11.6(5.71)			11.3(5.63)	11.6(5.71)		
**Basal FSH, IU/l**				0.021				-0.0873
Median [Q1,Q3]	6.89 [5.84,8.17]	6.77 [5.88,8.14]	0.581		7.07 [5.88,8.56]	6.77 [5.88,8.15]	0.0509	
Mean(SD)	7.21(2.25)	7.27(2.46)			7.48(2.38)	7.27(2.47)		
**Basal LH, IU/l**				-0.2209				0.0549
Median [Q1,Q3]	4.68 [3.48,6.27]	4.31 [3.36,5.62]	<0.001		4.37 [3.36,5.57]	4.30 [3.36,5.61]	0.839	
Mean(SD)	5.43(3.25)	4.87(2.55)			4.73(2.24)	4.87(2.55)		
**Basal PRL, ng/L**				0.0114				-0.0197
Median [Q1,Q3]	14.7 [10.8,20.3]	14.2 [9.88,19.8]	0.138		14.8 [10.9,21.1]	14.2 [9.88,19.8]	0.0935	
Mean(SD)	16.6(9.29)	16.8(22.2)			17.3(12.3)	16.8(22.2)		
**Tubal factor**				-0.0259				-0.0499
without	3643 (36.6%)	189 (39.2%)	0.269		165 (34.3%)	189 (39.3%)	0.124	
with	6306 (63.4%)	293 (60.8%)			316 (65.7%)	292 (60.7%)		
**Hysteromyoma**				0.0281				0.0062
without	9362 (94.1%)	440 (91.3%)	0.0148		442 (91.9%)	439 (91.3%)	0.816	
with	587 (5.9%)	42 (8.7%)			39 (8.1%)	42 (8.7%)		
**Uterine adhesion**				0.0254				-0.0042
without	9417 (94.7%)	444 (92.1%)	0.022		441 (91.7%)	443 (92.1%)	0.906	
with	532 (5.3%)	38 (7.9%)			40 (8.3%)	38 (7.9%)		
**PCOS**				-0.0286				0.0249
without	9087 (91.3%)	454 (94.2%)	0.035		465 (96.7%)	453 (94.2%)	0.0896	
with	862 (8.7%)	28 (5.8%)			16 (3.3%)	28 (5.8%)		
**Endometriosis**				0.0369				0.0021
without	9160 (92.1%)	426 (88.4%)	0.00492		426 (88.6%)	425 (88.4%)	>0.99	
with	789 (7.9%)	56 (11.6%)			55 (11.4%)	56 (11.6%)		
**Hysteroscopic abnormalities**				0.0601				0.0021
without	9226 (92.7%)	418 (86.7%)	<0.001		418 (86.9%)	417 (86.7%)	>0.99	
with	723 (7.3%)	64 (13.3%)			63 (13.1%)	64 (13.3%)		
**E 2 level on HCG day, ng/l**				-0.2081				0.015
Median [Q1,Q3]	4120 [2400,5910]	3590 [2120,4990]	<0.001		3460 [1910,4830]	3590 [2120,4990]	0.594	
Mean(SD)	4500 (2830)	3950 (2600)			3910 (2700)	3950 (2600)		
**Oocyte yield**				-0.2223				0.0299
Median [Q1,Q3]	11.0 [7.00,16.0]	10.0 [7.00,14.0]	<0.001		9.00 [6.00,14.0]	10.0 [7.00,14.0]	0.395	
Mean(SD)	11.8(6.35)	10.6(5.63)			10.4(5.97)	10.6(5.64)		
**Insemination method**								
ICSI	2615 (26.3%)	147 (30.5%)	0.00743	0.0421	141 (29.3%)	146 (30.4%)	0.89	0.0104
IVF	7286 (73.2%)	329 (68.3%)		-0.0498	335 (69.6%)	329 (68.4%)		-0.0125
IVF/ICSI	48 (0.5%)	6 (1.2%)		0.0076	5 (1.0%)	6 (1.2%)		0.0021
**Available Embryo number**				-0.2106				0.0394
Median [Q1,Q3]	6.00 [4.00,10.0]	6.00 [4.00,8.00]	<0.001		5.00 [3.00,8.00]	6.00 [4.00,8.00]	0.19	
Mean(SD)	7.13(4.23)	6.35(3.74)			6.20(4.12)	6.34(3.75)		
**Good morphology embryo transferred**								
0	1358 (13.6%)	82 (17.0%)	<0.001	0.0336	83 (17.3%)	81 (16.8%)	0.836	-0.0042
1	8320 (83.6%)	374 (77.6%)		-0.0603	368 (76.5%)	374 (77.8%)		0.0125
2	271 (2.7%)	26 (5.4%)		0.0267	30 (6.2%)	26 (5.4%)		-0.0083
**Embryo transfer cycle**								
1	2816 (28.3%)	26 (5.4%)	<0.001	-0.2291	26 (5.4%)	26 (5.4%)	0.978	0
2	4280 (43.0%)	185 (38.4%)		-0.0464	187 (38.9%)	185 (38.5%)		-0.0042
3	1785 (17.9%)	151 (31.3%)		0.1339	145 (30.1%)	151 (31.4%)		0.0125
>3	1068 (10.7%)	120 (24.9%)		0.1416	123 (25.6%)	119 (24.7%)		-0.0083
**Endometrial preparation**								
GnRHa+HRT	4362 (43.8%)	387 (80.3%)	<0.001	0.3645	391 (81.3%)	386 (80.2%)	0.991	-0.0104
HRT	1854 (18.6%)	39 (8.1%)		-0.1054	37 (7.7%)	39 (8.1%)		0.0042
OI	206 (2.1%)	11 (2.3%)		0.0021	11 (2.3%)	11 (2.3%)		0
Other	97 (1.0%)	4 (0.8%)		-0.0015	3 (0.6%)	4 (0.8%)		0.0021
NC	3430 (34.5%)	41 (8.5%)		-0.2597	39 (8.1%)	41 (8.5%)		0.0042
**Endometrial thickness, mm**				-0.1053				0.0351
Median [Q1,Q3]	8.90 [7.90,10.2]	8.70 [7.70,10.0]	0.0194		8.70 [7.60,10.0]	8.70 [7.70,10.0]	0.831	
Mean(SD)	9.15(1.86)	8.95(1.85)			8.89(1.79)	8.96(1.85)		
**Suboptimal endometrial pattern**				0.0136				0.0021
no	9321 (93.7%)	445 (92.3%)	0.271		445 (92.5%)	444 (92.3%)	>0.99	
yes	628 (6.3%)	37 (7.7%)			36 (7.5%)	37 (7.7%)		
**DTF**				-0.1501				-0.0368
Median [Q1,Q3]	0.900 [0.700,1.10]	0.800 [0.600,1.00]	<0.001		0.800 [0.700,1.00]	0.800 [0.600,1.00]	0.301	
Mean(SD)	0.891(0.305)	0.846(0.302)			0.858(0.271)	0.847(0.302)		
**Stage of embryo transferred**								
D3	1043 (10.5%)	30 (6.2%)	<0.001	-0.0426	40 (8.3%)	30 (6.2%)	0.391	-0.0208
D5	7386 (74.2%)	345 (71.6%)		-0.0266	329 (68.4%)	344 (71.5%)		0.0312
D6	1520 (15.3%)	107 (22.2%)		0.0629	112 (23.3%)	107 (22.2%)		-0.0104
**Number of embryos transferred**								
1	7124 (71.6%)	257 (53.3%)	<0.001	-0.1829	257 (53.4%)	256 (53.2%)	NA	-0.0021
2	2821 (28.4%)	225 (46.7%)		0.1833	224 (46.6%)	225 (46.8%)		0.0021

Data were presented as mean ± SD and median [first quartile, third quartile] for continuous variables and n (percentage) for categorical variables. *D: Standardized difference. The absolute value of D is less than 0.1, cohorts can be considered to be balanced concerning the demographics being assessed. PCOS, polycystic ovarian syndrome; FSH, follicle-stimulating hormone; LH, luteinizing hormone; PRL, prolactin; E2, estradiol; GnRHa, Gonadotropin-releasing hormone agonist; HRT, hormone replacement therapy; OI, ovulation promotion; NC, natural cycle; DTF, Distance of embryo transfer from uterine fundus.

**Table 2** shows the ET outcomes of patients receiving atosiban and GH as adjuvants alone or in combination. For all patients receiving atosiban administration, no obvious effect on pregnancy was observed in comparison with either matched or unmatched controls. However, when the patients were stratified according to GH administration, atosiban showed a significant association with clinical pregnancy in comparison with either matched or unmatched controls among patients with GH treatment with rate ratios (RR) of 1.32 (95%CI: 1.05,1.67) and 1.35 (95%CI: 1,1.82), respectively. On the other hand, however, the association was absent among patients without GH treatment. The descriptive characteristics of subgroups are shown in **Supplementary Table S1** and **Supplemenatary Table S2**. We also analyze the effect of GH treatment in a matched cohort based on GH treatment assignment (**Supplementary Figure S4**, **Supplementary Table S3**), finding an insignificant association.

**Table 2:** Clinical outcomes unmatched and matched cohort.

	Unmatched cohort			Matched cohort		
	Non-Atosiban	Atosiban	P-value	Non-Atosiban	Atosiban	P-value
All						
**N**	9949	482		481	481	
Pregnancy						
Prevalence	5677 (57.1%)	266 (55.2%)	0.445	249 (51.9%)	265 (55.2%)	0.332
RR (95%CI)	Ref	0.97(0.89,1.05)		Ref	1.06(0.95,1.2)	
Ectopic pregnancy						
Prevalence	39 (0.7%)	3 (1.1%)	0.642	0 (0%)	3 (1.1%)	0.269
RR (95%CI)	Ref	1.64(0.51,5.28)		Ref	NA	
Miscarriage						
Prevalence	691 (12.2%)	43 (16.2%)	0.0659	27 (10.8%)	43 (16.2%)	0.099
RR (95%CI)	Ref	1.33(1,1.76)		Ref	1.5(0.96,2.34)	
GH subgroup						
**N**	196	79		79	78	
Pregnancy						
Prevalence	90 (45.9%)	48 (60.8%)	0.0363	36 (45.6%)	48 (61.5%)	0.0649
RR (95%CI)	Ref	1.32(1.05,1.67)		Ref	1.35(1,1.82)	
Ectopic pregnancy						
Prevalence	1 (1.1%)	0 (0%)	1	0 (0%)	0 (0%)	NA
RR (95%CI)		NA		Ref	NA	
Miscarriage						
Prevalence	16 (17.8%)	11 (22.9%)	0.617	7 (19.4%)	11 (22.9%)	0.908
RR (95%CI)		1.29(0.65,2.55)		Ref	1.18(0.51,2.74)	
Non-GH subgroup						
**N**	9753	403		402	403	
Pregnancy						
Prevalence	5587 (57.3%)	218 (54.1%)	0.224	241 (60.0%)	218 (54.1%)	0.108
RR (95%CI)	Ref	0.94(0.86,1.03)		Ref	0.9(0.8,1.02)	
Ectopic pregnancy						
Prevalence	38 (0.7%)	3 (1.4%)	0.429	0 (0%)	3 (1.4%)	0.212
RR (95%CI)	Ref	2.02(0.63,6.53)		Ref	NA	
Miscarriage						
Prevalence	675 (12.1%)	32 (14.7%)	0.296	36 (14.9%)	32 (14.7%)	1
RR (95%CI)	Ref	1.21(0.86,1.71)		Ref	0.98(0.63,1.53)	

RR, rate ratio; CI, confidence interval.

**Figure 1:**
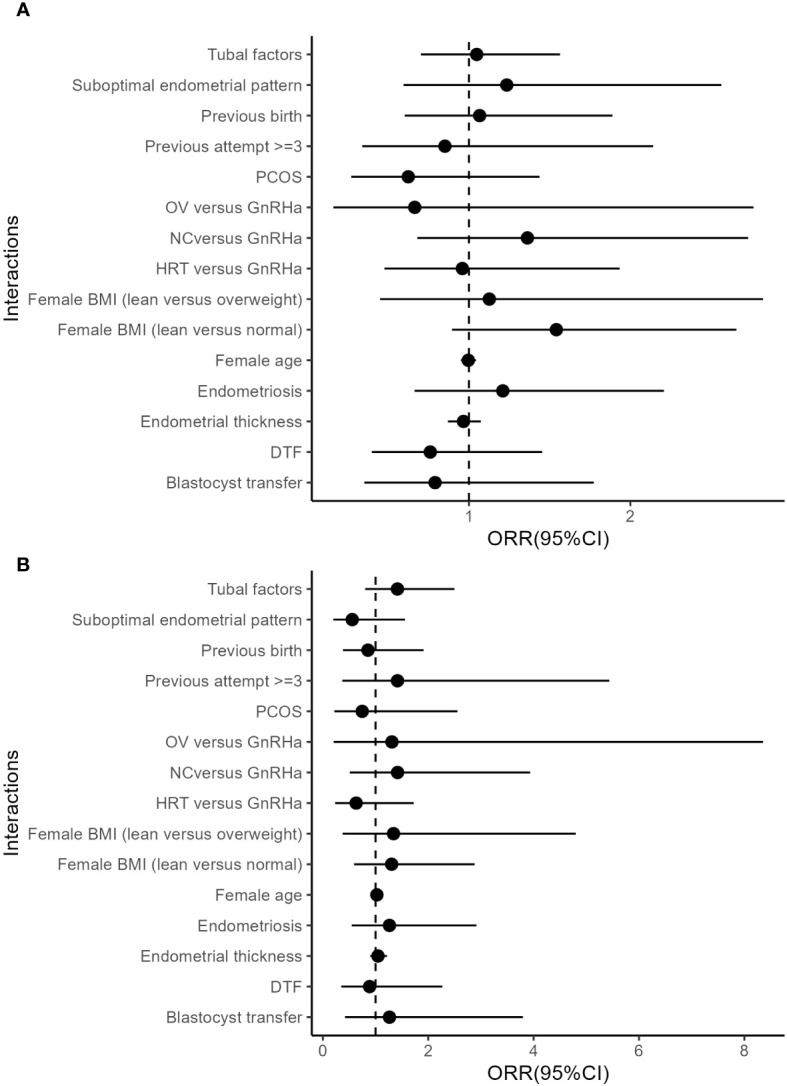
Interactions between atosiban and covariates. **(A)** Unmatched cohort. **(B)** Matched cohort. All models were adjusted for female, and male age, parity, basal FSH, LH, PRL, and AFC, diagnoses of tubal factor, hysteromyoma, uterine adhesion, PCOS, endometriosis, hysteroscopic abnormalities, and E 2 level on HCG day, oocyte yield, insemination method, available Embryo number and good morphology embryo transferred, embryo transfer order, endometrial preparation, endometrial thickness, suboptimal endometrial pattern, DTF, stage of embryo transferred, and the number of embryos transferred as independent variables.

The results of stratified multivariate analyses and interaction analyses are demonstrated in **Table 3**. The ORs for clinical pregnancy comparing patients with and without atosiban showed associations with opposite directions in patients with and without GH treatment. The adjusted ORRs in both matched and unmatched cohorts were 2.44 (95%CI: 1.07, 5.84) and 1.95 (95%CI: 1.05, 3.49) respectively, indicating a significant interaction. In addition to the interaction between atosiban and GH, we also tested a series of potential interactions with atosiban in the multivariate models, including the factors suggested by previous studies, such as age, endometriosis, and endometrial thickness. However, none of these interaction terms reached significance (**Figure 1**).

**Table 3:** The interaction between atosiban and GH.

		OR comparing Atosiban versus non-Atosiban (95% CI)	p-value	ORR (95%CI)	p-value
**Matched cohort**	**Non-GH subgroup**	0.87 (0.70, 1.08)	0.2	ref	ref
	**GH subgroup**	1.59 (0.85, 2.99)	0.15	1.91 (1.05, 3.49)	0.034
**Unmatched cohort**	**Non-GH subgroup**	0.74 (0.55, 1.01)	0.055	ref	ref
	**GH subgroup**	2.44 (1.07, 5.84)	0.038	2.29 (1.08, 4.88)	0.031

OR, odds ratio; CI, confidence interval; ORR, ratio of OR.

All models were adjusted for female, and male age, parity, basal FSH, LH, PRL, and AFC, diagnoses of tubal factor, hysteromyoma, uterine adhesion, PCOS, endometriosis, hysteroscopic abnormalities, and E 2 level on HCG day, oocyte yield, insemination method, available Embryo number and good morphology embryo transferred, embryo transfer order, endometrial preparation, endometrial thickness, suboptimal endometrial pattern, DTF, stage of embryo transferred, and number of embryos transferred as independent variables. The pregnancy rate was the dependent variable, and atosiban and GH are the interaction terms.

Subgroup analyses (**Supplementary Table S4**) showed that for patients with a thin endometrium or patients with previous ET attempts, atosiban alone had no significant association with pregnancy. However, the interaction between atosiban and GH remained significant in patients with a thin endometrium. The significance of the interaction diminished in patients with previous ET attempts, properly due to the reduced statistical power, but the figure still indicated a similar trend (P=0.1).

## Discussion

### Main finding

Our study suggested a possible combined effect of atosiban and GH as adjuvants which might benefit the clinical pregnancy. On the other hand, however, atosiban or GH showed no significant effect on pregnancy in the cohort. The finding may support the concept of “combination therapy” (16) to enhance the pregnancy outcome in FET cycles.

### Strengths and limitations

To the best of our knowledge, the study is the first to demonstrate the interaction between atosiban and GH in FET cycles. The study design is also fortified by the sample size and a control cohort matched for indications, such as previous ET attempts and endometriosis diagnosis. Nevertheless, the study is still limited by its retrospective nature, which may include unknown or unmeasured confounding or biases. In addition, the euploidy of the embryos transferred was not known in the study, which may significantly affect the measure of the effect.

The measurement of uterine contraction is also absent in the study. It led to an obscure indication and thus might affect the evaluation of the effect of atosiban. However, among the few studies that reported uterine contraction in cycles with atosiban treatment, the uterine peristalsis frequency may not always be relevant to the outcome (17).

In the present study, we used a total dose of 37.5mg atosiban in the study. According to the published data, there have been various types of doses and modes of administration (bolus versus infusion) of atosiban as an adjuvant in ART and they may contribute to the heterogeneity of the studies (6). Nevertheless, the reviews of previous data (6, 18) also suggested that the association between atosiban and ET outcomes is not necessarily linked to the dose of atosiban administration and the effect of different doses/modes of atosiban administration remained less clear.

### Interpretation

Although the use of adjuvants in ART appears to be common, the combination of various adjuvants is not always promising. For instance, Motteram et al. reported a combined adjuvant strategy of aspirin, doxycycline, and prednisolone, suggesting no benefit in fresh IVF cycles, and possible harm when used in frozen cycles (19). Low molecular weight heparin, aspirin, and prednisolone, another combination of adjuvants to enhance the outcomes in patients with previous implantation failure is also reported to have a neutral effect (20). Since the data concerning the effect of combined adjuvants to support embryo implantation remained limited, and a neutral even suboptimal outcome would be expected, a careful consultation may be needed. Our data may contribute to future consultation on the use of combined adjuvants.

A possible interpretation of the interactions between atosiban and GH is based on the cross-talk of the signals they mediate. Atosiban is an inhibitor of oxytocin used to reduce the frequency and amplitude of uterine contractions during ET. However, oxytocin has also been shown to have physiologic, metabolic, and anabolic effects targeting the GH-IGF1 axis apart from its role in smooth muscle contractions (21). The oxytocin receptor (OXTR) also interacts with the GH secretagogue (ghrelin) receptor, which consequently was able to attenuate OXTR-mediated Gaq signaling (22). Intravenous oxytocin administration is reported to reduce the circulating levels of ghrelin (23). Growth hormone, in turn, also stimulates the release of oxytocin in a dose-dependent manner (24). When GH is administrated during endometrial preparation with the hope of improving endometrial receptivity, the enhanced GH signaling might also enhance the oxytocin release, which might increase the risks of unwanted uterine contraction. Additional atosiban treatment, therefore might be helpful in these patients.

We did not find a significant association between atosiban treatment alone and pregnancy outcomes in either the overall population or in patients without GH treatment. It appeared to contract with the recent Cochrane review conclusion that intravenous atosiban may increase clinical pregnancy rate (RR 1.50, 95% CI 1.18 to 1.89) (18). However, the largest trial in the review (25) which enrolled 800 individuals suggested a neutral effect of atosiban on patients without specific indications. On the other hand, the smaller RCTs showed significant effects of atosiban may have aggregated indications, such as endometriosis (26) or difficulty in transfer (27). In addition, several more recent studies based on patients with RIF found no significant effect of atosiban on pregnancy rates (20, 28, 29). The evidence as a whole may be against the routine use of atosiban in unselected patients.

Since our atosiban patients were matched for both GH treatment and endometrial thickness, another interpretation for the significant effect of atosiban in GH treated cohort may be the aggregation of patients that have similar indications. It is reported that GH treatment may benefit patients with a thin endometrial thickness (30) and therefore clinicians may tend to use GH in patients with a thin endometrium. Our data also showed a lower mean endometrial thickness in GH-treated patients. On the other hand, however, a thin endometrial thickness is thought to be linked with abnormal uterine peristalsis (31). Atosiban might be more effective in those patients due to a potential bias of selection. Nevertheless, we screened the potential interactions between the atosiban and patients’ indications, including age, BMI, etiologies, and endometrial thickness without a significant finding (**Figure 1**). Therefore, it is yet to be concluded that endometrial thickness is a potential marker for atosiban use.

A summary of previous studies (30) suggested that GH as an adjuvant could improve ET outcomes by enhancing endometrial receptivity. However, the majority of evidence included in that review was focused on fresh cycles and biased toward poor responders. The potential effects of GH on oocytes and embryos could not be excluded. In FET cycles, where the quantity and quality of the embryos had been determined previously, a few reports also supported the role of GH in improving the endometrial thickness, blood perfusion, and receptivity markers (32, 33). However, the data may not justify the routine use of GH in FET cycles, as they were small and few in number, limited in patients with a thin endometrium. Our data demonstrated in a larger cohort with matched characteristics that GH may not significantly improve pregnancy rates in unselected patients. In body systems, GH is a pleiotropic hormone affecting multiple physiological systems, interacting with numerous signaling pathways including oxytocin. Moreover, evidence also suggested its potential pathological role (34). The complexity of the physiological role of the hormone suggests that the administration of GH during endometrial preparation simultaneously affects multiple tissues and potentially interacts with various internal or external factors. It may warrant further studies regarding the interactions between GH and physiological/therapeutic factors.

## Conclusions

The combination use of adjuvant therapies may be a viable option in the clinical practice of embryo transfer. However, evidence to support the practice remained limited. Interaction between adjuvants suggested that their effects were not simply added up. Our finding showed that the combination use of atosiban and GH in FET cycles is potentially beneficial or at least not detrimental to the pregnancy. On the other hand, however, indications for the use of atosiban and GH may need further assessment.

## Data availability statement

The raw data supporting the conclusions of this article will be made available by the authors, without undue reservation.

## Ethics statement

Institutional Review Board approval of this retrospective study was obtained from the Ethical Committee of the Xiamen University affiliated Chenggong hospital. The studies involving humans were approved by Ethical Committee of the Xiamen University affiliated Chenggong hospital. The studies were conducted in accordance with the local legislation and institutional requirements. Written informed consent for participation was not required from the participants or the participants' legal guardians/next of kin in accordance with the national legislation and institutional requirements.

## Author contributions

HC: Writing – original draft, Writing – review & editing, Conceptualization, Data curation, Formal analysis. JC: Conceptualization, Data curation, Formal analysis, Writing – original draft, Writing – review & editing. XS: Data curation, Investigation, Writing – review & editing. lL: Conceptualization, Data curation, Formal analysis, Writing – review & editing. ZL: Data curation, Investigation, Writing – review & editing. PG: Conceptualization, Data curation, Writing – review & editing. XJ: Conceptualization, Data curation, Supervision, Writing – review & editing. JR: Conceptualization, Funding acquisition, Supervision, Writing – review & editing.

## Supplementary material

Supplementary Figure 1 Flowchart of patient inclusion.

Supplementary Figure 2 Directed acyclic graphic for covariates. The green cycle indicates exposure of interest. The blue cycle indicates the outcome of interest. The white cycles indicate the adjusted covariates in the multivariate analyses. The grey cycle indicates an element that could not be measured directly.

Supplementary Figure 3 Distribution of propensity scores in patients with or without atosiban treatment in the unmatched and matched cohort.

Supplementary Figure 4 Distribution of propensity scores in patients with or without GH treatment in the unmatched and matched cohort.

## References

[1] Margalioth EJ Ben-Chetrit A Gal M Eldar-Geva T. Investigation and treatment of repeated implantation failure following IVF-ET. Hum Reprod (2006) 21:3036–43. doi: 10.1093/humrep/del305 16905766

[2] Tyler B Walford H Tamblyn J Keay SD Mavrelos D Yasmin E. Interventions to optimize embryo transfer in women undergoing assisted conception: a comprehensive systematic review and meta-analyses. Hum Reprod Update. (2022) 28:480–500. doi: 10.1093/humupd/dmac009 35325124 PMC9631462

[3] Jones GL Lang V Hudson N. A baby at all costs? Exploring the use and provision of unproven adjuvant treatments in the context of IVF. Semin Reprod Med. (2021) 39:220–6. doi: 10.1055/s-0041-1731789 34500475

[4] Human Fertilisation and Embryology Authority. National Patient Survey 2021 | HFEA (2024). Available online at: https://www.hfea.gov.uk/about-us/publications/research-and-data/national-patient-survey-2021/#treatment-add-ons.

[5] Lensen S Hammarberg K Polyakov A Wilkinson J Whyte S Peate M. How common is add-on use and how do patients decide whether to use them? A national survey of IVF patients. Hum Reprod (2021) 36:1854–61. doi: 10.1093/humrep/deab098 33942073

[6] Wang R Huang H Tan Y Xia G. Efficacy of atosiban for repeated embryo implantation failure: A systematic review and meta-analysis. Front Endocrinol. (2023) 14:1161707. doi: 10.3389/fendo.2023.1161707 PMC10076890 37033236

[7] Altmäe S Mendoza-Tesarik R Mendoza C Mendoza N Cucinelli F Tesarik J. Effect of growth hormone on uterine receptivity in women with repeated implantation failure in an oocyte donation program: A randomized controlled trial. J Endocr Soc. (2018) 2:96–105. doi: 10.1210/js.2017-00359 29379897 PMC5779111

[8] Sirotkin AV Nitray J. Growth hormone and prolactin affect oxytocin, vasopressin, progesterone and cyclic nucleotide secretion by bovine granulosa cells in vitro. J Endocrinol. (1994) 143:417–22. doi: 10.1677/joe.0.1430417 7836885

[9] Cai J Liu L Zhang J Qiu H Jiang X Li P. Low body mass index compromises live birth rate in fresh transfer in *vitro* fertilization cycles: a retrospective study in a Chinese population. Fertil Steril. (2017) 107:422–429.e2. doi: 10.1016/j.fertnstert.2016.10.029 27887711

[10] Alpha Scientists in Reproductive Medicine and ESHRE Special Interest Group of Embryology. The Istanbul consensus workshop on embryo assessment: proceedings of an expert meeting. Hum Reprod (2011) 26:1270–83. doi: 10.1093/humrep/der037 21502182

[11] Gardner DK Vella P Lane M Wagley L Schlenker T Schoolcraft WB. Culture and transfer of human blastocysts increases implantation rates and reduces the need for multiple embryo transfers. Fertil Steril. (1998) 69:84–8. doi: 10.1016/S0015-0282(97)00438-X 9457939

[12] Ho D Imai K King G Stuart EA. MatchIt: nonparametric preprocessing for parametric causal inference. J Stat Software. (2011) 42:1–28. doi: 10.18637/jss.v042.i08

[13] Greifer N. R package version 4.3.2. In: Cobalt: covariate balance tables and plots (2022). Available online at: https://CRAN.R-project.org/package=cobalt.

[14] Austin PC. An introduction to propensity score methods for reducing the effects of confounding in observational studies. Multivar Behav Res. (2011) 46:399–424. doi: 10.1080/00273171.2011.568786 PMC3144483 21818162

[15] Greifer N. cobalt: Covariate Balance Tables and Plots (2024). Available online at: https://cran.r-project.org/web/packages/cobalt/index.html.

[16] He Y Tang R Yu H Mu H Jin H Dong J. Comparative effectiveness and safety of 36 therapies or interventions for pregnancy outcomes with recurrent implantation failure: a systematic review and network meta-analysis. J Assist Reprod Genet. (2023) 40:2343–56. doi: 10.1007/s10815-023-02923-8 PMC10504168 37661207

[17] Buddhabunyakan N Sothornwit J Seejorn K Buppasiri P Salang L. Effects of atosiban on uterine peristalsis following frozen embryo transfer: A randomized controlled trial. Eur J Obstet Gynecol Reprod Biol. (2021) 265:96–101. doi: 10.1016/j.ejogrb.2021.08.017 34478926

[18] Craciunas L Tsampras N Kollmann M Raine-Fenning N Choudhary M. Oxytocin antagonists for assisted reproduction. Cochrane Database Syst Rev. (2021) 9:CD012375. doi: 10.1002/14651858.CD012375.pub2 34467530 PMC8408576

[19] Motteram C Vollenhoven B Hope N Osianlis T Rombauts LJ. Live birth rates after combined adjuvant therapy in IVF-ICSI cycles: a matched case-control study. Reprod BioMed Online. (2015) 30:340–8. doi: 10.1016/j.rbmo.2014.12.004 25676168

[20] Aslan K Kasapoglu I Cinar C Cakir C Avci B Uncu G. Low molecular weight heparin-aspirin-prednisolone combination does not increase the live birth rate in recurrent implantation failure: A retrospective cohort study. Reprod Sci. (2023) 30:3253–60. doi: 10.1007/s43032-023-01233-9 37253934

[21] Sohlström A Carlsson-Skwirut C Bang P Brismar K Uvnäs-Moberg K. Effects of oxytocin treatment early in pregnancy on fetal growth in ad libitum-fed and food-restricted rats. Pediatr Res. (1999) 46:339–44. doi: 10.1203/00006450-199909000-00016 10473052

[22] Borroto-Escuela DO Cuesta-Marti C Lopez-Salas A Chruścicka-Smaga B Crespo-Ramírez M Tesoro-Cruz E. The oxytocin receptor represents a key hub in the GPCR heteroreceptor network: potential relevance for brain and behavior. Front Mol Neurosci. (2022) 15:1055344. doi: 10.3389/fnmol.2022.1055344 36618821 PMC9812438

[23] Vila G Riedl M Resl M van der Lely AJ Hofland LJ Clodi M. Systemic administration of oxytocin reduces basal and lipopolysaccharide-induced ghrelin levels in healthy men. J Endocrinol. (2009) 203:175–9. doi: 10.1677/JOE-09-0227 19587265

[24] Sirotkin AV. Direct action of growth hormone on bovine ovarian cells: effects on estradiol, oxytocin, vasopressin release by granulosa cells and on oocyte maturation and cleavage in *vitro* . Ann Endocrinol. (1996) 57:219–24. 8949418

[25] Ng EHY Li RHW Chen L Lan VTN Tuong HM Quan S. A randomized double blind comparison of atosiban in patients undergoing IVF treatment. Hum Reprod (2014) 29:2687–94. doi: 10.1093/humrep/deu263 25336707

[26] He Y Wu H He X Xing Q Zhou P Cao Y. Administration of atosiban in patients with endometriosis undergoing frozen-thawed embryo transfer: a prospective, randomized study. Fertil Steril. (2016) 106:416–22. doi: 10.1016/j.fertnstert.2016.04.019 27143518

[27] Yuan C Song H Fan L Su S Dong B. The effect of atosiban on patients with difficult embryo transfers undergoing *in vitro* fertilization-embryo transfer. Reprod Sci. (2019) 26:1613–7. doi: 10.1177/1933719119831791 30791824

[28] Tang CL Li QY Chen FL Cai CT Dong YY Wu YY. A randomized double blind comparison of atosiban in patients with recurrent implantation failure undergoing IVF treatment. Reprod Biol Endocrinol. (2022) 20:124. doi: 10.1186/s12958-022-00999-y 35986323 PMC9389813

[29] Li X Du Y Han X Wang H Sheng Y Lian F. Efficacy of atosiban for repeated implantation failure in frozen embryo transfer cycles. Sci Rep. (2023) 13:9277. doi: 10.1038/s41598-023-36286-y 37286752 PMC10247710

[30] Shang Y Wu M He R Ye Y Sun X. Administration of growth hormone improves endometrial function in women undergoing in *vitro* fertilization: a systematic review and meta-analysis. Hum Reprod Update. (2022) 28(6):838–57. doi: 10.1093/humupd/dmac028 35641113

[31] Rombauts L McMaster R Motteram C Fernando S. Risk of ectopic pregnancy is linked to endometrial thickness in a retrospective cohort study of 8120 assisted reproduction technology cycles. Hum Reprod (2015) 30:2846–52. doi: 10.1093/humrep/dev249 26428211

[32] Xue-Mei W Hong J Wen-Xiang Z Yang L. The effects of growth hormone on clinical outcomes after frozen-thawed embryo transfer. Int J Gynaecol Obstet (2016) 133:347–50. doi: 10.1016/j.ijgo.2015.10.020 27101995

[33] Cui N Li AM Luo ZY Zhao ZM Xu YM Zhang J. Effects of growth hormone on pregnancy rates of patients with thin endometrium. J Endocrinol Invest. (2019) 42:27–35. doi: 10.1007/s40618-018-0877-1 29671256

[34] Liu FT Wu Z Yan J Norman RJ Li R. The potential role of growth hormone on the endometrium in assisted reproductive technology. Front Endocrinol. (2020) 11:49. doi: 10.3389/fendo.2020.00049 PMC7033614 32117072

