# Supplementary figures and images for: Atosiban interacts with growth hormones as adjuvants in frozen-thawed embryo transfer cycles
Source: Front Endocrinol (Lausanne). 2024 May 22;15:1380778. doi: 10.3389/fendo.2024.1380778 (PMC11150816; doi:10.3389/fendo.2024.1380778)

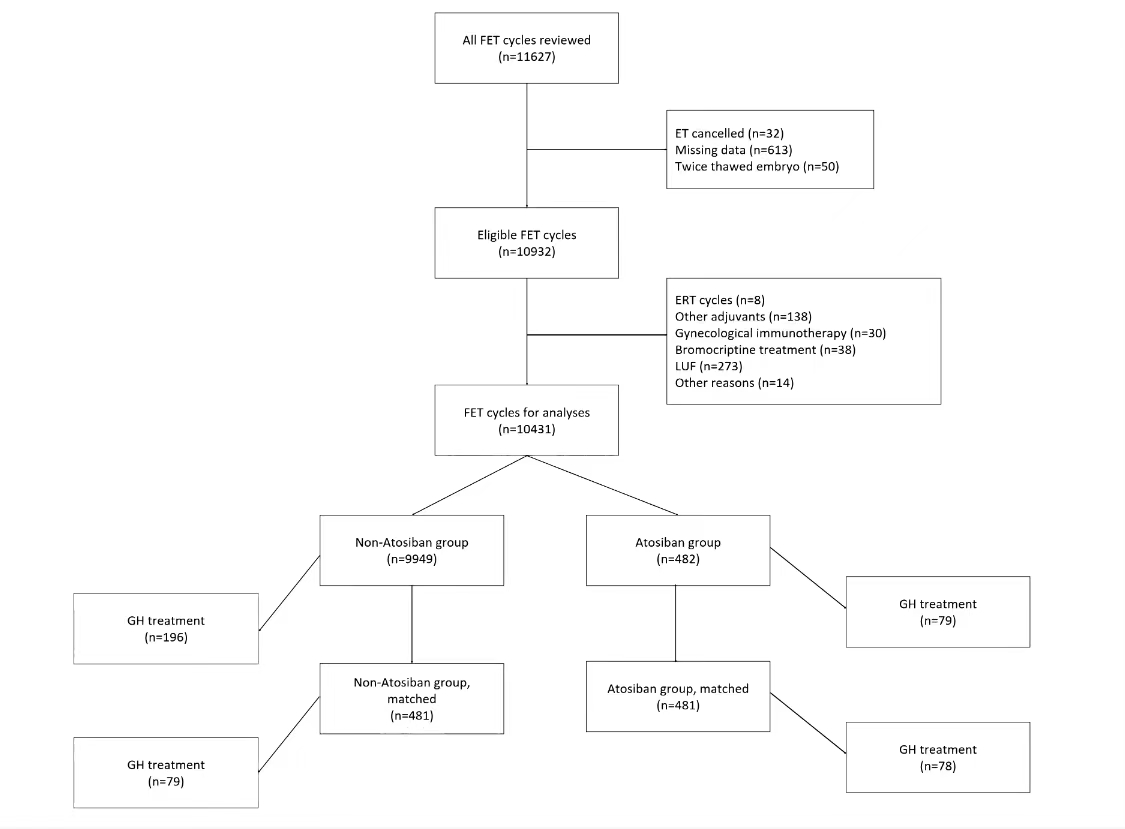

Supplement: Supplementary Figure 1 — Flowchart of patient inclusion. [file Image_1.jpeg]

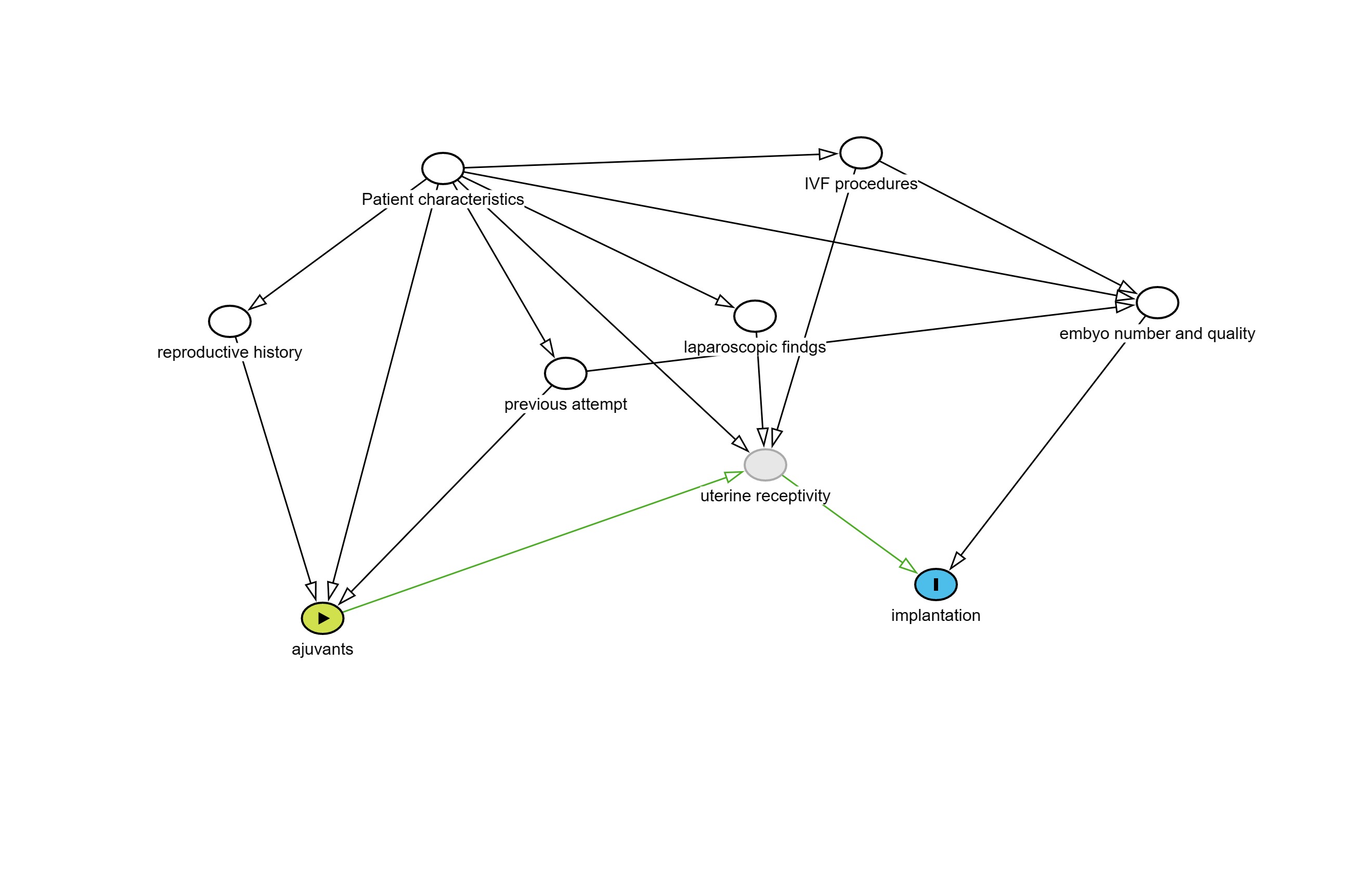

Supplement: Supplementary Figure 2 — Directed acyclic graphic for covariates. The green cycle indicates exposure of interest. The blue cycle indicates the outcome of interest. The white cycles indicate the adjusted covariates in the multivariate analyses. The grey cycle indicates an element that could not be measured directly. [file Image_2.jpeg]

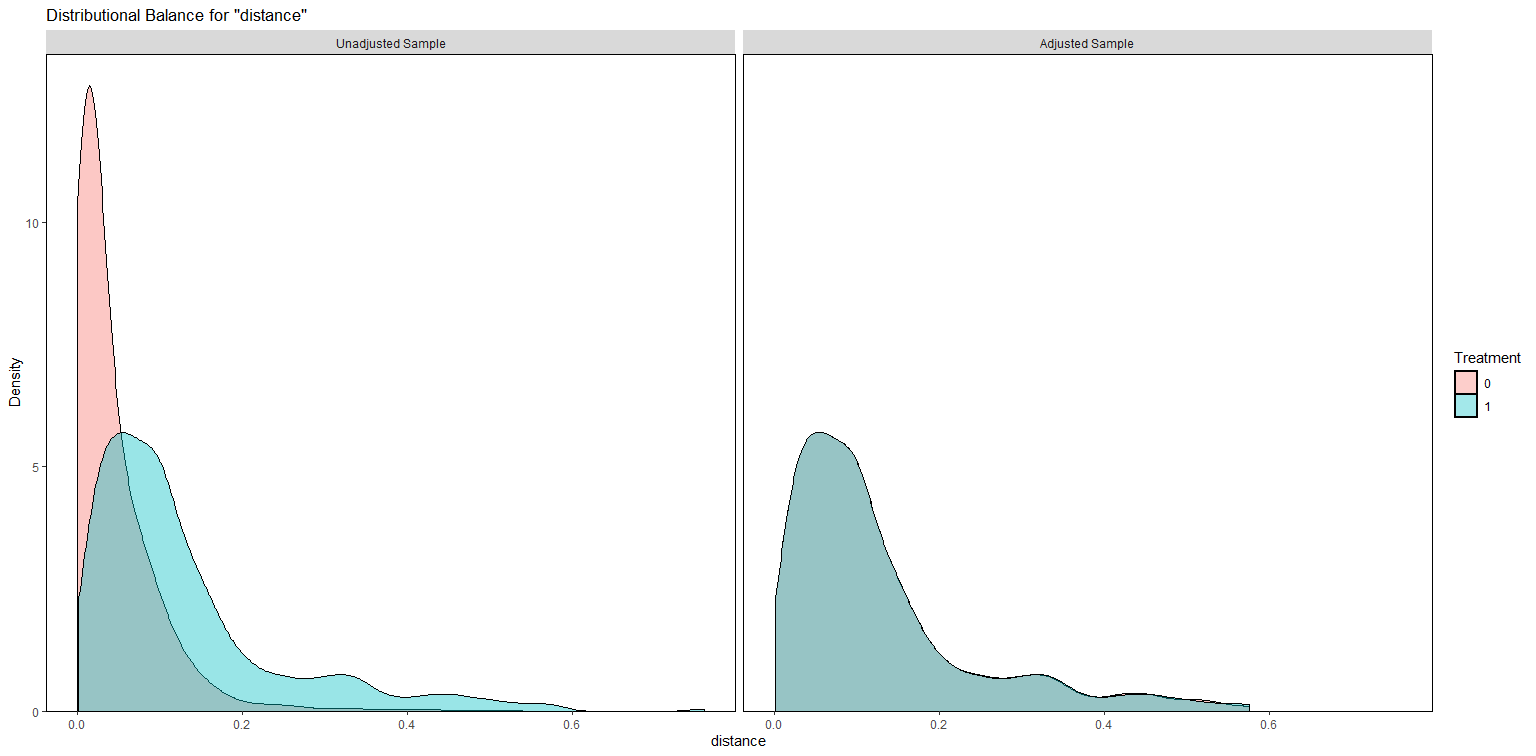

Supplement: Supplementary Figure 3 — Distribution of propensity scores in patients with or without atosiban treatment in the unmatched and matched cohort. [file Image_3.tif]

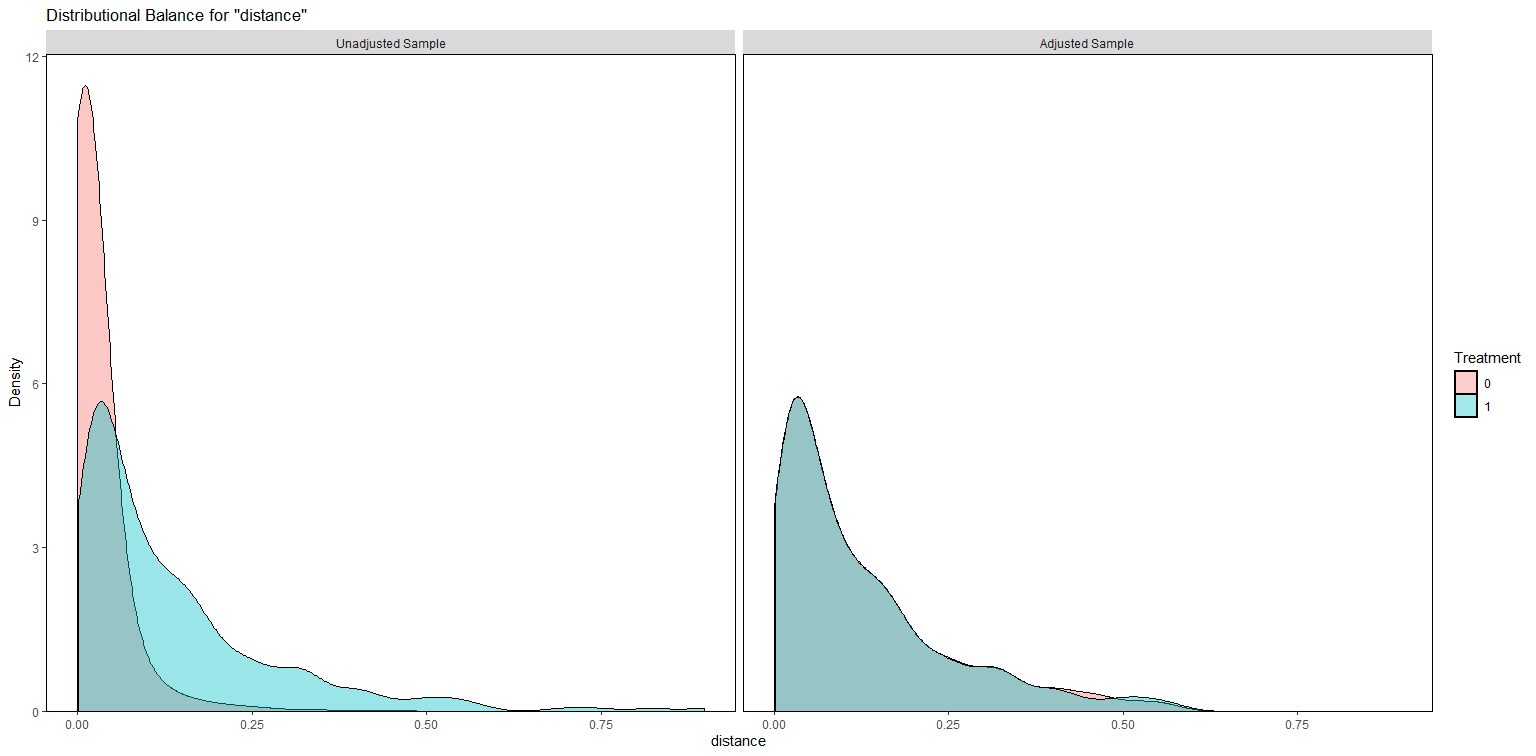

Supplement: Supplementary Figure 4 — Distribution of propensity scores in patients with or without GH treatment in the unmatched and matched cohort. [file Image_4.tif]
