# Supplementary material for: Atosiban interacts with growth hormones as adjuvants in frozen-thawed embryo transfer cycles
Source: Front Endocrinol (Lausanne). 2024 May 22;15:1380778. doi: 10.3389/fendo.2024.1380778 (PMC11150816; doi:10.3389/fendo.2024.1380778)
Supplement: Supplementary file 5 [file Table_1.docx]

Table S1 The descriptive characteristics of patients receiving GH

|  | **Unmatched** | | | **Matched** | | | |
| --- | --- | --- | --- | --- | --- | --- | --- |
|  | **Non- Atosiban** | **Atosiban** | **P-value** | **Non- Atosiban** | **Atosiban** | | **P-value** |
|  | **(N=196)** | **(N=79)** |  | **(N=79)** | **(N=78)** | |  |
| **Female age, yr** |  |  |  |  | |  |  |
| Median [Q1,Q3] | 32.0 [30.0,36.0] | 33.0 [31.0,36.0] | 0.259 | 33.0 [30.0,37.0] | | 33.0 [31.0,36.0] | 0.954 |
| Mean(SD) | 33.1(4.52) | 33.7(4.23) |  | 33.8(4.71) | | 33.6(4.22) |  |
| **Male age, yr** |  |  |  |  | |  |  |
| Median [Q1,Q3] | 34.0 [31.0,38.0] | 34.0 [31.5,37.0] | 0.997 | 34.0 [31.0,39.0] | | 34.0 [31.3,36.8] | 0.536 |
| Mean(SD) | 34.9(5.30) | 34.7(4.65) |  | 35.4(5.54) | | 34.6(4.67) |  |
| **Parity** |  |  |  |  | |  |  |
| 0 | 180 (91.8%) | 70 (88.6%) | 0.541 | 71 (89.9%) | | 69 (88.5%) | 0.978 |
| ≧1 | 16 (8.2%) | 9 (11.4%) |  | 8 (10.1%) | | 9 (11.5%) |  |
| **AFC** |  |  |  |  | |  |  |
| Median [Q1,Q3] | 11.0 [7.00,16.0] | 11.0 [8.00,16.0] | 0.704 | 11.0 [7.50,16.5] | | 11.0 [8.00,16.0] | 0.865 |
| Mean(SD) | 12.0(6.40) | 12.2(5.81) |  | 11.9(5.97) | | 12.2(5.85) |  |
| **Basal FSH, IU/l** |  |  |  |  | |  |  |
| Median [Q1,Q3] | 6.62 [5.52,8.20] | 7.01 [5.90,8.55] | 0.188 | 6.97 [5.60,8.47] | | 7.03 [5.91,8.56] | 0.391 |
| Mean(SD) | 7.19(2.53) | 7.45(2.18) |  | 7.32(2.37) | | 7.47(2.19) |  |
| **Basal LH, IU/l** |  |  |  |  | |  |  |
| Median [Q1,Q3] | 4.74 [3.53,6.35] | 4.47 [3.58,5.55] | 0.184 | 4.61 [3.45,5.80] | | 4.47 [3.55,5.49] | 0.639 |
| Mean(SD) | 5.29(2.61) | 4.94(2.64) |  | 4.91(2.03) | | 4.93(2.66) |  |
| **Basal PRL, ng/L** |  |  |  |  | |  |  |
| Median [Q1,Q3] | 14.6 [10.2,19.9] | 15.2 [10.9,18.7] | 0.86 | 13.4 [10.3,18.6] | | 15.1 [10.8,18.9] | 0.647 |
| Mean(SD) | 17.0(9.70) | 15.3(5.94) |  | 15.7(7.89) | | 15.2(5.97) |  |
| **Tubal factor** |  |  |  |  | |  |  |
| without | 58 (29.6%) | 34 (43.0%) | 0.0458 | 24 (30.4%) | | 34 (43.6%) | 0.121 |
| with | 138 (70.4%) | 45 (57.0%) |  | 55 (69.6%) | | 44 (56.4%) |  |
| **Hysteromyoma** |  |  |  |  | |  |  |
| without | 183 (93.4%) | 77 (97.5%) | 0.288 | 74 (93.7%) | | 76 (97.4%) | 0.45 |
| with | 13 (6.6%) | 2 (2.5%) |  | 5 (6.3%) | | 2 (2.6%) |  |
| **Uterine adhesion** |  |  |  |  | |  |  |
| without | 162 (82.7%) | 69 (87.3%) | 0.437 | 66 (83.5%) | | 68 (87.2%) | 0.676 |
| with | 34 (17.3%) | 10 (12.7%) |  | 13 (16.5%) | | 10 (12.8%) |  |
| **PCOS** |  |  |  |  | |  |  |
| without | 180 (91.8%) | 75 (94.9%) | 0.523 | 77 (97.5%) | | 74 (94.9%) | 0.666 |
| with | 16 (8.2%) | 4 (5.1%) |  | 2 (2.5%) | | 4 (5.1%) |  |
| **Endometriosis** |  |  |  |  | |  |  |
| without | 186 (94.9%) | 74 (93.7%) | 0.911 | 75 (94.9%) | | 73 (93.6%) | 0.984 |
| with | 10 (5.1%) | 5 (6.3%) |  | 4 (5.1%) | | 5 (6.4%) |  |
| **Hysteroscopic abnormalities** |  |  |  |  | |  |  |
| without | 164 (83.7%) | 64 (81.0%) | 0.724 | 67 (84.8%) | | 63 (80.8%) | 0.646 |
| with | 32 (16.3%) | 15 (19.0%) |  | 12 (15.2%) | | 15 (19.2%) |  |
| **E 2 level on HCG day, ng/l** |  |  |  |  | |  |  |
| Median [Q1,Q3] | 3440 [1890,4940] | 3740 [2370,5060] | 0.593 | 3460 [2050,4770] | | 3670 [2370,5030] | 0.76 |
| Mean(SD) | 3970(2670) | 4090(2600) |  | 3920(2350) | | 4050(2590) |  |
| **Oocyte yield** |  |  |  |  | |  |  |
| Median [Q1,Q3] | 10.0 [6.00,15.0] | 10.0 [7.50,14.5] | 0.478 | 9.00 [6.00,14.5] | | 10.0 [7.25,14.8] | 0.564 |
| Mean(SD) | 10.6(6.64) | 10.7(5.24) |  | 10.3(5.75) | | 10.7(5.27) |  |
| **Insemination method** |  |  |  |  | |  |  |
| ICSI | 49 (25.0%) | 24 (30.4%) | 0.64 | 25 (31.6%) | | 23 (29.5%) | 0.958 |
| IVF | 145 (74.0%) | 54 (68.4%) |  | 53 (67.1%) | | 54 (69.2%) |  |
| IVF/ICSI | 2 (1.0%) | 1 (1.3%) |  | 1 (1.3%) | | 1 (1.3%) |  |
| **Available Embryo number** |  |  |  |  | |  |  |
| Median [Q1,Q3] | 6.00 [3.00,8.00] | 7.00 [4.00,8.00] | 0.244 | 6.00 [3.00,8.50] | | 6.50 [4.00,8.00] | 0.233 |
| Mean(SD) | 6.28(4.21) | 6.49(3.34) |  | 5.97(3.91) | | 6.47(3.35) |  |
| **Good morphology embryo transferred** |  |  |  |  | |  |  |
| 0 | 35 (17.9%) | 13 (16.5%) | 0.834 | 15 (19.0%) | | 12 (15.4%) | 0.619 |
| 1 | 156 (79.6%) | 63 (79.7%) |  | 59 (74.7%) | | 63 (80.8%) |  |
| 2 | 5 (2.6%) | 3 (3.8%) |  | 5 (6.3%) | | 3 (3.8%) |  |
| **Embryo transfer order** |  |  |  |  | |  |  |
| 1 | 25 (12.8%) | 2 (2.5%) | 0.0661 | 4 (5.1%) | | 2 (2.6%) | 0.283 |
| 2 | 64 (32.7%) | 29 (36.7%) |  | 21 (26.6%) | | 29 (37.2%) |  |
| 3 | 58 (29.6%) | 29 (36.7%) |  | 27 (34.2%) | | 29 (37.2%) |  |
| **＞3** | 49 (25.0%) | 19 (24.1%) |  | 27 (34.2%) | | 18 (23.1%) |  |
| **Endometrial**  **preparation** |  |  |  |  | |  |  |
| GnRHa+HRT | 159 (81.1%) | 75 (94.9%) | 0.026 | 74 (93.7%) | | 74 (94.9%) | 0.301 |
| HRT | 25 (12.8%) | 4 (5.1%) |  | 2 (2.5%) | | 4 (5.1%) |  |
| OI | 10 (5.1%) | 0 (0%) |  | 2 (2.5%) | | 0 (0%) |  |
| NC | 2 (1.0%) | 0 (0%) |  | 1 (1.3%) | | 0 (0%) |  |
| **Endometrial thickness, mm** |  |  |  |  | |  |  |
| Median [Q1,Q3] | 7.80 [6.70,8.93] | 8.00 [7.15,9.45] | 0.0315 | 7.90 [6.85,9.25] | | 8.00 [7.23,9.48] | 0.275 |
| Mean(SD) | 7.95(1.81) | 8.52(1.91) |  | 8.14(1.78) | | 8.54(1.92) |  |
| **Suboptimal endometrial pattern** |  |  |  |  | |  |  |
| no | 174 (88.8%) | 73 (92.4%) | 0.496 | 71 (89.9%) | | 72 (92.3%) | 0.799 |
| yes | 22 (11.2%) | 6 (7.6%) |  | 8 (10.1%) | | 6 (7.7%) |  |
| **DTF** |  |  |  |  | |  |  |
| Median [Q1,Q3] | 0.800 [0.600,1.00] | 0.800 [0.600,1.00] | 0.918 | 0.800 [0.650,1.00] | | 0.800 [0.600,1.00] | 0.669 |
| Mean(SD) | 0.815(0.250) | 0.835(0.331) |  | 0.833(0.254) | | 0.839(0.331) |  |
| **Stage of embryo transferred** |  |  |  |  | |  |  |
| D3 | 23 (11.7%) | 5 (6.3%) | 0.381 | 10 (12.7%) | | 5 (6.4%) | 0.381 |
| D5 | 130 (66.3%) | 54 (68.4%) |  | 48 (60.8%) | | 53 (67.9%) |  |
| D6 | 43 (21.9%) | 20 (25.3%) |  | 21 (26.6%) | | 20 (25.6%) |  |
| **Number of embryos transferred** |  |  |  |  | |  |  |
| 1 | 128 (65.3%) | 40 (50.6%) | 0.024 | 44 (55.7%) | | 39 (50.0%) | 0.475 |
| 2 | 68 (34.7%) | 39 (49.4%) |  | 35 (44.3%) | | 39 (50.0%) |  |

Data were presented as mean ± SD and median [first quartile, third quartile] for continuous variables and n (percentage) for categorical variables. *D: Standardized difference. The absolute value of D is less than 0.1, cohorts can be considered to be balanced concerning the demographics being assessed. PCOS, polycystic ovarian syndrome; FSH, follicle-stimulating hormone; LH, luteinizing hormone; PRL, prolactin; E2, estradiol; GnRHa, Gonadotropin-releasing hormone agonist; HRT, hormone replacement therapy; OI, ovulation promotion; NC, natural cycle; DTF, Distance of embryo transfer from uterine fundus.
