# Supplementary material for: Atosiban interacts with growth hormones as adjuvants in frozen-thawed embryo transfer cycles
Source: Front Endocrinol (Lausanne). 2024 May 22;15:1380778. doi: 10.3389/fendo.2024.1380778 (PMC11150816; doi:10.3389/fendo.2024.1380778)
Supplement: Supplementary file 6 [file Table_2.docx]

Table S2 The descriptive characteristics of patients without GH treatment

|  | | **non-GH Unmatched** | | | | | **non-GH matched** | | | | |
| --- | --- | --- | --- | --- | --- | --- | --- | --- | --- | --- | --- |
|  |  | **Non- Atosiban** | **Atosiban** | | **P-value** | | **Non- Atosiban** | | **Atosiban** | **P-value** | |
|  | | **(N=9753)** | **(N=403)** | |  | | **(N=402)** | | **(N=403)** |  | |
| **Female age，yr** |  | | |  |  |  | |  | |  |  |
| Median [Q1,Q3] | 32.0 [29.0,35.0] | | | 32.0 [30.0,35.0] | 0.138 | 32.0 [30.0,36.0] | | 32.0 [30.0,35.0] | | 0.566 |  |
| Mean(SD) | 32.2(4.18) | | | 32.6(4.28) |  | 32.8(4.28) | | 32.6(4.28) | |  |  |
| **Male age,yr** |  | | |  |  |  | |  | |  |  |
| Median [Q1,Q3] | 33.0 [30.0,36.0] | | | 34.0 [31.0,37.0] | 0.0118 | 34.0 [31.0,37.0] | | 34.0 [31.0,37.0] | | 0.64 |  |
| Mean(SD) | 33.8(4.72) | | | 34.4(4.68) |  | 34.4(4.63) | | 34.4(4.68) | |  |  |
| **Parity** |  | | |  |  |  | |  | |  |  |
| 0 | 8252 (84.6%) | | | 348 (86.4%) | 0.378 | 346 (86.1%) | | 348 (86.4%) | | 0.989 |  |
| ≧1 | 1501 (15.4%) | | | 55 (13.6%) |  | 56 (13.9%) | | 55 (13.6%) | |  |  |
| **AFC** |  | | |  |  |  | |  | |  |  |
| Median [Q1,Q3] | 11.0 [8.00,16.0] | | | 11.0 [7.00,15.0] | 0.0724 | 11.0 [7.00,15.0] | | 11.0 [7.00,15.0] | | 0.73 |  |
| Mean(SD) | 12.0(6.17) | | | 11.5(5.69) |  | 11.2(5.56) | | 11.5(5.69) | |  |  |
| **Basal FSH, IU/l** |  | | |  |  |  | |  | |  |  |
| Median [Q1,Q3] | 6.89 [5.84,8.16] | | | 6.73 [5.85,8.05] | 0.3 | 7.15 [5.95,8.55] | | 6.73 [5.85,8.05] | | 0.0122 |  |
| Mean(SD) | 7.22(2.24) | | | 7.23(2.52) |  | 7.52(2.38) | | 7.23(2.52) | |  |  |
| **Basal LH, IU/l** |  | | |  |  |  | |  | |  |  |
| Median [Q1,Q3] | 4.68 [3.48,6.27] | | | 4.28 [3.33,5.66] | <0.001 | 4.34 [3.33,5.56] | | 4.28 [3.33,5.66] | | 0.692 |  |
| Mean(SD) | 5.44(3.26) | | | 4.86(2.53) |  | 4.70(2.28) | | 4.86(2.53) | |  |  |
| **Basal PRL, ng/L** |  | | |  |  |  | |  | |  |  |
| Median [Q1,Q3] | 14.7 [10.8,20.3] | | | 13.8 [9.83,19.9] | 0.135 | 15.1 [10.9,21.5] | | 13.8 [9.83,19.9] | | 0.0482 |  |
| Mean(SD) | 16.6(9.28) | | | 17.2(24.2) |  | 17.6(13.0) | | 17.2(24.2) | |  |  |
| **Tubal factor** |  | | |  |  |  | |  | |  |  |
| without | 3585 (36.8%) | | | 155 (38.5%) | 0.521 | 141 (35.1%) | | 155 (38.5%) | | 0.356 |  |
| with | 6168 (63.2%) | | | 248 (61.5%) |  | 261 (64.9%) | | 248 (61.5%) | |  |  |
| **Hysteromyoma** |  | | |  |  |  | |  | |  |  |
| without | 9179 (94.1%) | | | 363 (90.1%) | 0.00125 | 368 (91.5%) | | 363 (90.1%) | | 0.549 |  |
| with | 574 (5.9%) | | | 40 (9.9%) |  | 34 (8.5%) | | 40 (9.9%) | |  |  |
| **Uterine adhesion** |  | | |  |  |  | |  | |  |  |
| without | 9255 (94.9%) | | | 375 (93.1%) | 0.128 | 375 (93.3%) | | 375 (93.1%) | | 1 |  |
| with | 498 (5.1%) | | | 28 (6.9%) |  | 27 (6.7%) | | 28 (6.9%) | |  |  |
| **PCOS** |  | | |  |  |  | |  | |  |  |
| without | 8907 (91.3%) | | | 379 (94.0%) | 0.0687 | 388 (96.5%) | | 379 (94.0%) | | 0.137 |  |
| with | 846 (8.7%) | | | 24 (6.0%) |  | 14 (3.5%) | | 24 (6.0%) | |  |  |
| **Endometriosis** |  | | |  |  |  | |  | |  |  |
| without | 8974 (92.0%) | | | 352 (87.3%) | 0.00112 | 351 (87.3%) | | 352 (87.3%) | | 1 |  |
| with | 779 (8.0%) | | | 51 (12.7%) |  | 51 (12.7%) | | 51 (12.7%) | |  |  |
| **Hysteroscopic abnormalities** |  | | |  |  |  | |  | |  |  |
| without | 9062 (92.9%) | | | 354 (87.8%) | <0.001 | 351 (87.3%) | | 354 (87.8%) | | 0.904 |  |
| with | 691 (7.1%) | | | 49 (12.2%) |  | 51 (12.7%) | | 49 (12.2%) | |  |  |
| **E 2 level on HCG day,ng/l** |  | | |  |  |  | |  | |  |  |
| Median [Q1,Q3] | 4130 [2400,5920] | | | 3530 [2110,4940] | <0.001 | 3470 [1910,4830] | | 3530 [2110,4940] | | 0.638 |  |
| Mean(SD) | 4510(2830) | | | 3930(2600) |  | 3910(2770) | | 3930(2600) | |  |  |
| **Oocyte yield** |  | | |  |  |  | |  | |  |  |
| Median [Q1,Q3] | 11.0 [7.00,16.0] | | | 10.0 [6.00,14.0] | <0.001 | 9.00 [6.00,14.0] | | 10.0 [6.00,14.0] | | 0.521 |  |
| Mean(SD) | 11.9(6.34) | | | 10.6(5.71) |  | 10.5(6.02) | | 10.6(5.71) | |  |  |
| **Insemination method** |  | | |  |  |  | |  | |  |  |
| ICSI | 2566 (26.3%) | | | 123 (30.5%) | 0.0145 | 116 (28.9%) | | 123 (30.5%) | | 0.818 |  |
| IVF | 7141 (73.2%) | | | 275 (68.2%) |  | 282 (70.1%) | | 275 (68.2%) | |  |  |
| IVF/ICSI | 46 (0.5%) | | | 5 (1.2%) |  | 4 (1.0%) | | 5 (1.2%) | |  |  |
| **Available Embryo number** |  | | |  |  |  | |  | |  |  |
| Median [Q1,Q3] | 7.00 [4.00,10.0] | | | 6.00 [3.00,8.00] | <0.001 | 5.00 [3.00,8.00] | | 6.00 [3.00,8.00] | | 0.369 |  |
| Mean(SD) | 7.15(4.23) | | | 6.32(3.82) |  | 6.24(4.16) | | 6.32(3.82) | |  |  |
| **Good morphology embryo transferred** |  | | |  |  |  | |  | |  |  |
| 0 | 1323 (13.6%) | | | 69 (17.1%) | <0.001 | 68 (16.9%) | | 69 (17.1%) | | 0.953 |  |
| 1 | 8164 (83.7%) | | | 311 (77.2%) |  | 309 (76.9%) | | 311 (77.2%) | |  |  |
| 2 | 266 (2.7%) | | | 23 (5.7%) |  | 25 (6.2%) | | 23 (5.7%) | |  |  |
| **Embryo transfer cycle** |  | | |  |  |  | |  | |  |  |
| 1 | 2791 (28.6%) | | | 24 (6.0%) | <0.001 | 22 (5.5%) | | 24 (6.0%) | | 0.899 |  |
| 2 | 4216 (43.2%) | | | 156 (38.7%) |  | 166 (41.3%) | | 156 (38.7%) | |  |  |
| 3 | 1727 (17.7%) | | | 122 (30.3%) |  | 118 (29.4%) | | 122 (30.3%) | |  |  |
| ＞3 | 1019 (10.4%) | | | 101 (25.1%) |  | 96 (23.9%) | | 101 (25.1%) | |  |  |
| **Endometrial preparation** |  | | |  |  |  | |  | |  |  |
| GnRHa+HRT | 4203 (43.1%) | | | 312 (77.4%) | <0.001 | 317 (78.9%) | | 312 (77.4%) | | 0.974 |  |
| HRT | 1829 (18.8%) | | | 35 (8.7%) |  | 35 (8.7%) | | 35 (8.7%) | |  |  |
| OI | 196 (2.0%) | | | 11 (2.7%) |  | 9 (2.2%) | | 11 (2.7%) | |  |  |
| Other | 97 (1.0%) | | | 4 (1.0%) |  | 3 (0.7%) | | 4 (1.0%) | |  |  |
| NC | 3428 (35.1%) | | | 41 (10.2%) |  | 38 (9.5%) | | 41 (10.2%) | |  |  |
| **Endometrial thickness, mm** |  | | |  |  |  | |  | |  |  |
| Median [Q1,Q3] | 8.90 [7.90,10.2] | | | 8.80 [7.80,10.1] | 0.227 | 8.90 [7.80,10.1] | | 8.80 [7.80,10.1] | | 0.832 |  |
| Mean(SD) | 9.17(1.85) | | | 9.04(1.82) |  | 9.04(1.75) | | 9.04(1.82) | |  |  |
| **Suboptimal endometrial pattern** |  | | |  |  |  | |  | |  |  |
| no | 9147 (93.8%) | | | 372 (92.3%) | 0.273 | 374 (93.0%) | | 372 (92.3%) | | 0.794 |  |
| yes | 606 (6.2%) | | | 31 (7.7%) |  | 28 (7.0%) | | 31 (7.7%) | |  |  |
| **DTF** |  | | |  |  |  | |  | |  |  |
| Median [Q1,Q3] | 0.900 [0.700,1.10] | | | 0.800 [0.630,1.00] | 0.00228 | 0.800 [0.700,1.00] | | 0.800 [0.630,1.00] | | 0.346 |  |
| Mean(SD) | 0.893(0.305) | | | 0.848(0.297) |  | 0.863(0.274) | | 0.848(0.297) | |  |  |
| **Stage of embryo transferred** |  | | |  |  |  | |  | |  |  |
| D3 | 1020 (10.5%) | | | 25 (6.2%) | <0.001 | 30 (7.5%) | | 25 (6.2%) | | 0.698 |  |
| D5 | 7256 (74.4%) | | | 291 (72.2%) |  | 281 (69.9%) | | 291 (72.2%) | |  |  |
| D6 | 1477 (15.1%) | | | 87 (21.6%) |  | 91 (22.6%) | | 87 (21.6%) | |  |  |
| **Number of embryos transferred** |  | | |  |  |  | |  | |  |  |
| 1 | 6996 (71.7%) | | | 217 (53.8%) | <0.001 | 213 (53.0%) | | 217 (53.8%) | | 0.807 |  |
| 2 | 2753 (28.2%) | | | 186 (46.2%) |  | 189 (47.0%) | | 186 (46.2%) | |  |  |

Data were presented as mean ± SD and median [first quartile, third quartile] for continuous variables and n (percentage) for categorical variables. *D: Standardized difference. The absolute value of D is less than 0.1, cohorts can be considered to be balanced concerning the demographics being assessed. PCOS, polycystic ovarian syndrome; FSH, follicle-stimulating hormone; LH, luteinizing hormone; PRL, prolactin; E2, estradiol; GnRHa, Gonadotropin-releasing hormone agonist; HRT, hormone replacement therapy; OI, ovulation promotion; NC, natural cycle; DTF, Distance of embryo transfer from uterine fundus.
