# Supplementary material for: Atosiban interacts with growth hormones as adjuvants in frozen-thawed embryo transfer cycles
Source: Front Endocrinol (Lausanne). 2024 May 22;15:1380778. doi: 10.3389/fendo.2024.1380778 (PMC11150816; doi:10.3389/fendo.2024.1380778)
Supplement: Supplementary file 7 [file Table_3.docx]

Table S3

|  | | | **Unmatched** | | | | | | **matched** | | | | |
| --- | --- | --- | --- | --- | --- | --- | --- | --- | --- | --- | --- | --- | --- |
|  |  |  | **Non-GH** | **GH** | | **P-value** | | ***D** | **Non-GH** | **GH** | **P-value** | ***D** | |
|  | | | **(N=10156)** | **(N=275)** | |  | |  | **(N=271)** | **(N=271)** |  |  | |
| **Female age,yr** |  | | |  |  | 0.2409 | |  |  |  | | 0.0632 |  |
| Median [Q1,Q3] | 32.0 [29.0,35.0] | | | 33.0 [30.0,36.0] | <0.001 |  | | 32.0 [30.0,36.0] | 32.0 [30.0,36.0] | 0.479 | |  |  |
| Mean(SD) | 32.2(4.19) | | | 33.3(4.44) |  |  | | 33.0(4.55) | 33.2(4.43) |  | |  |  |
| **Male age,yr** |  | | |  |  | 0.2023 | |  |  |  | | 0.0483 |  |
| Median [Q1,Q3] | 33.0 [30.0,36.3] | | | 34.0 [31.0,37.0] | 0.0029 |  | | 34.0 [31.0,37.5] | 34.0 [31.0,37.0] | 0.787 | |  |  |
| Mean(SD) | 33.8(4.72) | | | 34.8(5.11) |  |  | | 34.5(4.88) | 34.8(5.13) |  | |  |  |
| **Parity** |  | | |  |  | -0.0623 | |  |  |  | | -0.0111 |  |
| 0 | 8600 (84.7%) | | | 250 (90.9%) | 0.0058 |  | | 243 (89.7%) | 246 (90.8%) | 0.772 | |  |  |
| ≧1 | 1556 (15.3%) | | | 25 (9.1%) |  |  | | 28 (10.3%) | 25 (9.2%) |  | |  |  |
| **AFC** |  | | |  |  | 0.0084 | |  |  |  | | 0.0581 |  |
| Median [Q1,Q3] | 11.0 [8.00,16.0] | | | 11.0 [7.50,16.0] | 0.713 |  | | 11.0 [8.00,15.0] | 11.0 [7.50,16.0] | 0.93 | |  |  |
| Mean(SD) | 12.0(6.16) | | | 12.0(6.23) |  |  | | 11.6(5.52) | 12.0(6.20) |  | |  |  |
| **Basal FSH, IU/l** |  | | |  |  | 0.0193 | |  |  |  | | 0.0082 |  |
| Median [Q1,Q3] | 6.88 [5.84,8.16] | | | 6.80 [5.73,8.27] | 0.681 |  | | 6.91 [5.79,8.20] | 6.80 [5.72,8.31] | 0.778 | |  |  |
| Mean(SD) | 7.22(2.25) | | | 7.26(2.43) |  |  | | 7.25(2.13) | 7.27(2.45) |  | |  |  |
| **Basal LH, IU/l** |  | | |  |  | -0.0868 | |  |  |  | | 0.0523 |  |
| Median [Q1,Q3] | 4.66 [3.48,6.26] | | | 4.65 [3.54,6.12] | 0.912 |  | | 4.47 [3.34,5.82] | 4.64 [3.50,6.12] | 0.346 | |  |  |
| Mean(SD) | 5.41(3.23) | | | 5.19(2.62) |  |  | | 5.01(2.42) | 5.15(2.56) |  | |  |  |
| **Basal PRL, ng/L** |  | | |  |  | -0.0133 | |  |  |  | | -0.0554 |  |
| Median [Q1,Q3] | 14.7 [10.8,20.3] | | | 14.6 [10.2,19.6] | 0.831 |  | | 14.4 [10.9,20.6] | 14.6 [10.3,19.7] | 0.688 | |  |  |
| Mean(SD) | 16.6(10.3) | | | 16.5(8.81) |  |  | | 17.0(10.6) | 16.5(8.84) |  | |  |  |
| **Tubal factor** |  | | |  |  | -0.0111 | |  |  |  | | -0.0111 |  |
| without | 3740 (36.8%) | | | 92 (33.5%) | 0.28 |  | | 88 (32.5%) | 91 (33.6%) | 0.855 | |  |  |
| with | 6416 (63.2%) | | | 183 (66.5%) |  |  | | 183 (67.5%) | 180 (66.4%) |  | |  |  |
| **Hysteromyoma** |  | | |  |  | -0.0059 | |  |  |  | | 0.0037 |  |
| without | 9542 (94.0%) | | | 260 (94.5%) | 0.781 |  | | 257 (94.8%) | 256 (94.5%) | 1 | |  |  |
| with | 614 (6.0%) | | | 15 (5.5%) |  |  | | 14 (5.2%) | 15 (5.5%) |  | |  |  |
| **Uterine adhesion** |  | | |  |  | 0.1082 | |  |  |  | | 0.0037 |  |
| without | 9630 (94.8%) | | | 231 (84.0%) | <0.001 |  | | 229 (84.5%) | 228 (84.1%) | 1 | |  |  |
| with | 526 (5.2%) | | | 44 (16.0%) |  |  | | 42 (15.5%) | 43 (15.9%) |  | |  |  |
| **PCOS** |  | | |  |  | -0.0129 | |  |  |  | | 0.0148 |  |
| without | 9286 (91.4%) | | | 255 (92.7%) | 0.517 |  | | 256 (94.5%) | 252 (93.0%) | 0.595 | |  |  |
| with | 870 (8.6%) | | | 20 (7.3%) |  |  | | 15 (5.5%) | 19 (7.0%) |  | |  |  |
| **Endometriosis** |  | | |  |  | -0.0272 | |  |  |  | | -0.0111 |  |
| without | 9326 (91.8%) | | | 260 (94.5%) | 0.129 |  | | 253 (93.4%) | 256 (94.5%) | 0.719 | |  |  |
| with | 830 (8.2%) | | | 15 (5.5%) |  |  | | 18 (6.6%) | 15 (5.5%) |  | |  |  |
| **Hysteroscopic abnormalities** |  | | |  |  | 0.098 | |  |  |  | |  |  |
| without | 9416 (92.7%) | | | 228 (82.9%) | <0.001 |  | | 239 (88.2%) | 226 (83.4%) | 0.14 | |  |  |
| with | 740 (7.3%) | | | 47 (17.1%) |  |  | | 32 (11.8%) | 45 (16.6%) |  | |  |  |
| **E 2 level on HCG day,ng/l** |  | | |  |  | -0.182 | |  |  |  | | 0.0038 |  |
| Median [Q1,Q3] | 4110 [2390,5890] | | | 3590 [2000,5010] | 0.00112 |  | | 3540 [2270,5070] | 3590 [2000,5010] | 0.781 | |  |  |
| Mean(SD) | 4480(2830) | | | 4000(2640) |  |  | | 3990(2550) | 4000(2650) |  | |  |  |
| **Oocyte yield** |  | | |  |  | -0.183 | |  |  |  | | 0.0407 |  |
| Median [Q1,Q3] | 11.0 [7.00,16.0] | | | 10.0 [6.00,15.0] | 0.00176 |  | | 10.0 [6.00,14.0] | 10.0 [6.00,15.0] | 0.83 | |  |  |
| Mean(SD) | 11.8(6.33) | | | 10.7(6.26) |  |  | | 10.4(5.56) | 10.7(6.27) |  | |  |  |
| **Insemination method** |  | | |  |  |  | |  |  |  | |  |  |
| ICSI | 2689 (26.5%) | | | 73 (26.5%) | 0.405 | 0.0007 | | 67 (24.7%) | 72 (26.6%) | 0.886 | | 0.0185 |  |
| IVF | 7416 (73.0%) | | | 199 (72.4%) |  | -0.0066 | | 202 (74.5%) | 197 (72.7%) |  | | -0.0185 |  |
| IVF/ICSI | 51 (0.5%) | | | 3 (1.1%) |  | 0.0059 | | 2 (0.7%) | 2 (0.7%) |  | | 0 |  |
| **Available Embryo number** |  | | |  |  | -0.1956 | |  |  |  | | 0.0242 |  |
| Median [Q1,Q3] | 6.00 [4.00,10.0] | | | 6.00 [3.00,8.00] | 0.00206 |  | | 6.00 [3.00,8.00] | 6.00 [3.00,8.00] | 0.812 | |  |  |
| Mean(SD) | 7.12(4.22) | | | 6.34(3.97) |  |  | | 6.25(3.87) | 6.34(3.98) |  | |  |  |
| **Good morphology**  **embryo**  **transferred** |  | | |  |  |  | |  |  |  | |  |  |
| 0 | 1392 (13.7%) | | | 48 (17.5%) | 0.202 | 0.0375 | | 52 (19.2%) | 47 (17.3%) | 0.856 | | -0.0185 |  |
| 1 | 8475 (83.4%) | | | 219 (79.6%) |  | -0.0381 | | 211 (77.9%) | 216 (79.7%) |  | | 0.0185 |  |
| 2 | 289 (2.8%) | | | 8 (2.9%) |  | 0.0006 | | 8 (3.0%) | 8 (3.0%) |  | | 0 |  |
| **Embryo transfer order** |  | | |  |  |  | |  |  |  | |  |  |
| 1 | 2815 (27.7%) | | | 27 (9.8%) | <0.001 | -0.0179 | | 27 (10.0%) | 27 (10.0%) | 0.626 | | 0 |  |
| 2 | 4372 (43.0%) | | | 93 (33.8%) |  | -0.0923 | | 93 (34.3%) | 92 (33.9%) |  | | -0.0037 |  |
| 3 | 1849 (18.2%) | | | 87 (31.6%) |  | 0.1343 | | 73 (26.9%) | 85 (31.4%) |  | | 0.0443 |  |
| **＞3** | 1120 (11.0%) | | | 68 (24.7%) |  | 0.137 | | 78 (28.8%) | 67 (24.7%) |  | | -0.0406 |  |
| **Endometrial preparation** |  | | |  |  |  | |  |  |  | |  |  |
| GnRHa+HRT | 4515 (44.5%) | | | 234 (85.1%) | <0.001 | 0.4063 | | 227 (83.8%) | 230 (84.9%) | 0.774 | | 0.0111 |  |
| HRT | 1864 (18.4%) | | | 29 (10.5%) |  | -0.0781 | | 35 (12.9%) | 29 (10.7%) |  | | -0.0221 |  |
| OI | 207 (2.0%) | | | 10 (3.6%) |  | 0.016 | | 7 (2.6%) | 10 (3.7%) |  | | 0.0111 |  |
| OTHER | 101 (1.0%) | | | 0 (0%) |  | -0.0099 | | 0 (0%) | 0 (0%) |  | | 0 |  |
| NC | 3469 (34.2%) | | | 2 (0.7%) |  | 0.3343 | | 2 (0.7%) | 2 (0.7%) |  | | 0 |  |
| **Endometrial thickness, mm** |  | | |  |  | -0.5965 | |  |  |  | | 0.0145 |  |
| Median [Q1,Q3] | 8.90 [7.90,10.2] | | | 7.90 [6.80,9.10] | <0.001 |  | | 8.00 [7.00,9.00] | 7.90 [6.80,9.10] | 0.543 | |  |  |
| Mean(SD) | 9.17(1.85) | | | 8.11(1.85) |  |  | | 8.10(1.57) | 8.13(1.86) |  | |  |  |
| **Suboptimal endometrial pattern** |  | | |  |  | 0.0391 | |  |  |  | | 0.048 |  |
| no | 9519 (93.7%) | | | 247 (89.8%) | 0.0126 |  | | 256 (94.5%) | 243 (89.7%) | 0.0565 | |  |  |
| yes | 637 (6.3%) | | | 28 (10.2%) |  |  | | 15 (5.5%) | 28 (10.3%) |  | |  |  |
| **DTF** |  | | |  |  | -0.2571 | |  |  |  | | 0.0378 |  |
| Median [Q1,Q3] | 0.900 [0.700,1.10] | | | 0.800 [0.600,1.00] | <0.001 |  | | 0.800 [0.600,1.00] | 0.800 [0.600,1.00] | 0.514 | |  |  |
| Mean(SD) | 0.891(0.305) | | | 0.820(0.275) |  |  | | 0.808(0.290) | 0.819(0.276) |  | |  |  |
| **Stage of embryo transferred** |  | | |  |  |  | |  |  |  | |  |  |
| D3 | 1045 (10.3%) | | | 28 (10.2%) | 0.0029 | -0.0011 | | 24 (8.9%) | 27 (10.0%) | 0.694 | | 0.0111 |  |
| D5 | 7547 (74.3%) | | | 184 (66.9%) |  | -0.074 | | 177 (65.3%) | 182 (67.2%) |  | | 0.0185 |  |
| D6 | 1564 (15.4%) | | | 63 (22.9%) |  | 0.0751 | | 70 (25.8%) | 62 (22.9%) |  | | -0.0295 |  |
| **Number of embryos transferred** |  | | |  |  |  | | 257 (53.4%) | 256 (53.2%) | NA | |  |  |
| 1 | 7213 (71.0%) | | | 168 (61.1%) | 0.0015 | -0.0993 | | 171 (63.1%) | 166 (61.3%) | NA | | -0.0185 |  |
| 2 | 2939 (28.9%) | | | 107 (38.9%) |  | 0.0997 | | 100 (36.9%) | 105 (38.7%) |  | | 0.0185 |  |
| **pregancy** |  | | |  |  |  | |  |  |  | |  |  |
| no | 4351 (42.8%) | | | 137 (49.8%) | 0.0248 |  | | 128 (47.2%) | 134 (49.4%) | 0.667 | |  |  |
| yes | 5805 (57.2%) | | | 138 (50.2%) |  |  | | 143 (52.8%) | 137 (50.6%) |  | |  |  |
| **Adjusted OR for pregnancy*** | ref | | | 0.93(0.71, 1.21) | 0.6 |  | | ref | 1.01(0.69, 1.48) | >0.9 | |  |  |

Data were presented as mean ± SD and median [first quartile, third quartile] for continuous variables and n (percentage) for categorical variables. *D: Standardized difference. The absolute value of D is less than 0.1, cohorts can be considered to be balanced concerning the demographics being assessed. PCOS, polycystic ovarian syndrome; FSH, follicle-stimulating hormone; LH, luteinizing hormone; PRL, prolactin; E2, estradiol; GnRHa, Gonadotropin-releasing hormone agonist; HRT, hormone replacement therapy, OI, ovulation promotion; NC, natural cycle; DTF, Distance of embryo transfer from uterine fundus.

* models were adjusted for female, and male age, parity, basal FSH, LH, PRL, and AFC, diagnoses of tubal factor, hysteromyoma, uterine adhesion, PCOS, endometriosis, hysteroscopic abnormalities, and E 2 level on HCG day, oocyte yield, insemination method, available Embryo number and good morphology embryo transferred, embryo transfer order, endometrial preparation, endometrial thickness, suboptimal endometrial pattern, DTF, stage of embryo transferred, and number of embryos transferred as independent variables. Pregnancy rate was the dependent variable, and GH and atosiban were the interaction terms.
