# Supplementary material for: Atosiban interacts with growth hormones as adjuvants in frozen-thawed embryo transfer cycles
Source: Front Endocrinol (Lausanne). 2024 May 22;15:1380778. doi: 10.3389/fendo.2024.1380778 (PMC11150816; doi:10.3389/fendo.2024.1380778)
Supplement: Supplementary file 8 [file Table_4.docx]

| **Subgroup** | **non-Atosiban pregnancy** | **Atosiban pregnancy** | **RR(95%CI)** | **aOR (95%)** | **ORR (95%CI)** |
| --- | --- | --- | --- | --- | --- |
| **Endometrial thickness<8mm** | |  |  |  |  |
| Overall | 1361/2630(51.7%) | 84/149(56.4%) | 1.09(0.94,1.26) | 1.2(0.84,1.73) | - |
| GH | 42/104(40.4%) | 24/37(64.9%) | **1.61(1.15,2.24)** | **3.94(1.35,11.5)** | **2.54(1.02,6.35)** |
| non-GH | 1319/2526(52.2%) | 60/112(53.6%) | 1.03(0.86,1.22) | 1.01(0.67,1.53) | ref |
|  |  |  |  |  |  |
| **Previous ET attempt≧3** | |  |  |  |  |
| Overall | 1495/2853(52.4%) | 149/271(55.0%) | 1.05(0.94,1.18) | 1.07(0.81,1.4) | - |
| GH | 50/107(46.7%) | 30/48(62.5%) | 1.34(0.99,1.8) | 2.02(0.81,5.05) | 1.93(0.87,4.26) |
| non-GH | 1445/2746(52.6%) | 119/223(53.4%) | 1.01(0.89,1.15) | 0.96(0.72,1.3) | ref |

Table S4 Subgroup analyses for atosiban-growth hormone interactions in patients with thin endometrium and patients with previous implantation failure

Bold figures indicate significant at P<0.05 level.

aORs are adjusted odds ratios for pregnancy adjusted for female, and male age, parity, basal FSH, LH, PRL, and AFC, diagnoses of tubal factor, hysteromyoma, uterine adhesion, PCOS, endometriosis, hysteroscopic abnormalities, and E 2 level on HCG day, oocyte yield, insemination method, available Embryo number and good morphology embryo transferred, embryo transfer order, endometrial preparation, endometrial thickness, suboptimal endometrial pattern, DTF, stage of embryo transferred, and number of embryos transferred as independent variables. The pregnancy rate was the dependent variable, and atosiban and GH are the interaction terms.
